# Simulation of Ti-6Al-4V Additive Manufacturing Using Coupled Physically Based Flow Stress and Metallurgical Model

**DOI:** 10.3390/ma12233844

**Published:** 2019-11-21

**Authors:** Bijish Babu, Andreas Lundbäck, Lars-Erik Lindgren

**Affiliations:** 1 Swerim AB, Heating and Metalworking Box 812, SE-971 25 Luleå, Sweden; 2 Mechanics of Sold Materials, Luleå University of Technology, SE-971 87 Luleå, Sweden; Andreas.Lundback@ltu.se (A.L.); Lars-Erik.Lindgren@ltu.se (L.-E.L.)

**Keywords:** dislocation density, vacancy concentration, Ti-6Al-4V, additive manufacturing, directed energy deposition

## Abstract

Simulating the additive manufacturing process of Ti-6Al-4V is very complex due to the microstructural changes and allotropic transformation occurring during its thermomechanical processing. The α-phase with a hexagonal close pack structure is present in three different forms—Widmanstatten, grain boundary and Martensite. A metallurgical model that computes the formation and dissolution of each of these phases was used here. Furthermore, a physically based flow-stress model coupled with the metallurgical model was applied in the simulation of an additive manufacturing case using the directed energy-deposition method. The result from the metallurgical model explicitly affects the mechanical properties in the flow-stress model. Validation of the thermal and mechanical model was performed by comparing the simulation results with measurements available in the literature, which showed good agreement.

## 1. Introduction

Powder Bed Fusion (PBF) is the technique of building thin layer over layer by melting the fine metal powder. Directed energy deposition (DED), on the other hand, is usually used for building features on large existing parts as well as for repairing damaged ones. PBF typically adds layers that are thinner than DED and can therefore create high-resolution structures, whereas DED produces components at a higher built rate. The primary challenge of DED is that the higher energy input from the heat source may lead to substantial distortion and higher residual stresses.

DED additive manufacturing (AM) can be considered as computer numerically controlled (CNC) multipass welding with progressive weldments made on a substrate to create free-form structures. The added metals can be in either powder or wire form and the heat source a laser or electron beam. The deposition path is generated from computer-aided design (CAD) geometry and is preprogrammed in a CNC machine, which makes the process very flexible and suitable for low volume production, eliminating the need for tooling and dies.This also enables the production of complicated geometries that are traditionally difficult to produce with conventional manufacturing processes. Additively manufactured parts of Ti-6Al-4V are traditionally found in human implants [1] and aerospace components because of the criticality of their applications. However, AM has also been used to repair aerospace components [2] that have developed defects during operation or production.

A few researchers have performed AM simulations or similar processes for Ti alloys using thermomechanical–microstructural (TMM) coupled material models. In Baykasoglu et al. [3], a thermomicrostructural model for Ti6Al-4V was presented and applied to a DED process. Salsi et al. [4] presented a similar model and applied it on a PBF process, while Vastola et al. [5] compared the results when modelling electron-beam melting (EBM) and PBF processes. Song et al. [6] performed a welding simulation by using a TA15 alloy employing a TMM model. A similar model was utilized for performing a quenching simulation by Teixeira et al. [7] for alloy Til7. Cao et al. [8] showed an AM simulation using electron-beam melting without including microstructural coupling. A TMM material model was employed by Ahn et al. [9] for welding simulation ignoring strain-rate dependence.

In this work, a material model combining metallurgical and flow-stress models described by Babu et al. [10] is used. This model works for arbitrary phase composition and is an improved version of that of Babu and Lindgren [11]. The AM process involves cyclic heating and cooling, resulting in nonequilibrium phase evolution, which can be addressed with this model. The metallurgical model used in this work was also utilized in the simulation of the AM case described by Charles Murgau et al. [12], which is included in the current special issue.

## 2. Physically Based Flow-Stress Model

An incompressible von Mises model was used here with the assumption of isotropic plasticity. Flow stress was split into two parts [11,13,14,15]:

(1) $$\sigma_{y}=\sigma_{G}+\sigma^{*}.$$

Here, $\sigma_{G}$ is a thermal stress contribution from long-range interactions of the dislocation substructure. The other term, $\sigma^{*}$, is the required friction stress to move dislocations within the lattice and to cross short-range barriers. Thermal vibrations can assist dislocations to overcome these barriers. Conrad [16] proposed a similar formulation after analyzing titanium systems.

### 2.1. Long-Range Stress Component

The long-range term from Equation (1) is derived from Seeger [13] as

(2) $$\sigma_{G}=m\alpha Gb\sqrt{\rho_{i}}.$$

Here, *m* is the Taylor factor that translates the resolved shear stress in various slip systems to effective stress, *b* is the magnitude of Burgers vector, G(T) is the temperature-dependent shear modulus, $\rho_{i}$ is the immobile dislocation density and α(T) is a calibrated proportionality factor.

### 2.2. Short-Range Stress Component

The strain-rate-dependent part of the yield stress from Equation (1) can be derived according to the Kocks–Mecking formulation [17,18] as

(3) $$\sigma^{*}=\tau_{0}G{\left[1-{\left[\frac{kT}{\Delta f_{0}Gb^{3}}\ln\left(\frac{{\dot{\varepsilon }}^{ref}}{{\dot{\bar{\varepsilon }}}^{p}}\right)\right]}^{1/q}\right]}^{1/p}.$$

Here, shear strength in the absence of thermal energy is denoted by $\tau_{0}G$, and the activation energy required to overcome lattice resistance is denoted by $\Delta f_{0}Gb^{3}$. Parameters *p* and *q* define the shape of the obstacle barrier for dislocation motion. Further, *k* is the Boltzmann constant, *T* is the temperature in kelvin and (${\dot{\varepsilon }}^{ref}$) and (${\dot{\varepsilon }}^{p}$) are the reference and plastic strain rates, respectively.

### 2.3. Evolution of Immobile Dislocation Density

The evolution of $\rho_{i}$ in Equation (2) is modelled as having two components, hardening and restoration.

(4) $$\dot{\rho_{i}}={\dot{\rho_{i}}}^{\left(+\right)}-{\dot{\rho_{i}}}^{\left(-\right)}.$$

#### 2.3.1. Hardening Process

The average distance moved by dislocations before they are annihilated or immobilized is called mean free path Λ. The Orowan equation shows that the density of dislocations and their average velocity are proportional to the plastic strain rate. Assuming that the immobile dislocation density also follows the same relation leads to

(5) $${\dot{\rho_{i}}}^{\left(+\right)}=\frac{m}{b}\frac{1}{\Lambda }{\dot{\bar{\varepsilon }}}^{p}.$$

The mean free path is computed from grain size (*g*) and dislocation subcell or subgrain diameter (*s*) as

(6) $$\frac{1}{\Lambda }=\frac{1}{g}+\frac{1}{s}.$$

The subcell formation and evolution are modelled using a relation proposed by Holt [19].

(7) $$s=K_{c}\frac{1}{\sqrt{\rho_{i}}}.$$

#### 2.3.2. Restoration Processes

Vacancy motion is relevant to the recovery of dislocations. Restoration of the lattice commonly occurs at elevated temperatures and is therefore a thermally activated restructuring process. Creation of vacancy requires energy and increases entropy. With increasing temperature and deformation, vacancy concentration also increases. High stacking fault materials usually exhibit constant flow stress because of the balance between hardening and recovery. The current model assumes that the mechanisms of restoration are dislocation glide, dislocation climb and globularization.

(8) $${\dot{\rho_{i}}}^{\left(-\right)}={\dot{\rho_{i}}}^{\left(glide\right)}+{\dot{\rho_{i}}}^{\left(climb\right)}+{\dot{\rho_{i}}}^{\left(globularization\right)}.$$

The model for recovery by glide can be written on the basis of the formulation by Bergström [20] as

(9) $${\dot{\rho_{i}}}^{\left(glide\right)}=\Omega \rho_{i}{\dot{\bar{\varepsilon }}}^{p},$$

where Ω is a function dependent on temperature.

Militzer et al. [21] proposed a model for dislocation climb on the basis of Sandström and Lagneborg [22] and Mecking and Estrin [23]. With a modification of diffusivity according to Reference [11], the model can be written as

(10) $${\dot{\rho }}_{i}^{\left(climb\right)}=2c_{\gamma }D_{app}\frac{Gb^{3}}{kT}\left(\rho_{i}^{2}-\rho_{eq}^{2}\right),$$

where $c_{\gamma }$ is a material coefficient and $\rho_{eq}$ is the equilibrium value of the dislocation density. Here, $D_{app}$ is the apparent diffusivity that includes the diffusivity of the α−β phases weighted by their fractions $X_{\alpha }$ and $X_{\beta }$, pipe diffusion $D_{p}$, as well as effects of excess vacancy concentration $c_{v}$.

Babu and Lindgren [11] proposed a model for the evolution of dislocation density during globularization where the effect of grain growth on the reduction of flow stress is only included when dislocation density is above a critical value $\rho_{cr}$.

(11) $$\begin{array}{cc} \mathrm{if} & \rho_{i}\geq \rho_{cr}\\ & {\dot{\rho_{i}}}^{\left(globularization\right)}=\psi \dot{X_{g}}\left(\rho_{i}-\rho_{eq}\right);\;\;\mathrm{until}\;\rho_{i}\leq \rho_{eq} \end{array}$$

(12) $$\begin{array}{cc} \mathrm{else} & \\ & {\dot{\rho_{i}}}^{\left(globularization\right)}=0. \end{array}$$

Here, $\rho_{eq}$ is the equilibrium value of dislocation density, $\dot{X_{g}}$ is the globularization rate and ψ is a calibration constant. Thomas and Semiatin [24] modelled the two-stage process of dynamic and static recrystallization. Owing to the similarities between globularization and recrystallization, this model can be adapted.

(13) $$\begin{array}{ccc} X_{g} & = & X_{d}+\left(1-X_{d}\right)X_{s}. \end{array}$$

Here, volume fractions $X_{g}$, $X_{d}$, and $X_{s}$ denote total globularization, its dynamic component and the static component, respectively.

Assuming that grain growth and static recrystallization have the same driving force, the static globularization rate can be modelled as [25,26]

(14) $$\begin{array}{ccc} {\dot{X}}_{s} & = & M\frac{\dot{g}}{g}, \end{array}$$

where, *M* is a material parameter. The rate of dynamic globularization was computed on the basis of a model by Thomas and Semiatin [24] as,

(15) $$\begin{array}{ccc} {\dot{X}}_{d} & = & \frac{Bk_{p}\dot{\bar{\varepsilon_{p}}}}{{\bar{\varepsilon_{p}}}^{1-k_{p}}e^{B{\bar{\varepsilon }}_{p}^{k_{p}}}}, \end{array}$$

where, *B* and $k_{p}$ are material parameters.

### 2.4. Evolution of Excess Vacancy Concentration

The formation and evolution of excess vacancy concentration was modelled by Militzer et al. [21]. In the current work, Militzer’s model was extended by adding the effect of temperature changes. Further, assuming that only long-range stress contributes to vacancy formation, the model can be rewritten as

(16) $$\begin{array}{cc} {\dot{c_{v}}}^{ex}= & \left[\chi \frac{m\alpha Gb^{2}\sqrt{\rho_{i}}}{Q_{vf}}+\zeta \frac{c_{j}}{4b^{2}}\right]\frac{\Omega_{0}}{b}\dot{\bar{\varepsilon }}-D_{vm}\left[\frac{1}{s^{2}}+\frac{1}{g^{2}}\right]\left(c_{v}-c_{v}^{eq}\right)\\ & +c_{v}^{eq}\left(\frac{Q_{vf}}{kT^{2}}\right)\dot{T}. \end{array}$$

Here, χ=0.1 is the fraction of mechanical energy spent on vacancy generation, $\Omega_{0}$ is the atomic volume and ζ is the neutralization effect by vacancy emitting and absorbing jogs. The concentration of jogs ($c_{j}$) and $D_{vm}$ and the diffusivity of vacancy are given in Babu and Lindgren [11]. Additionally, $Q_{vf}$ is the activation energy of vacancy formation.

## 3. Phase-Evolution Model

A simplified model [27] for the transition between the liquid and solid state was implemented to take care of temperatures above melting temperature $T_{melt}$. If the temperature is above $T_{melt}$, the volume fraction of the solid phases was set to zero. In the solid state, the Ti–6Al–4V microstructure comprises the high-temperature stable β-phase and the lower-temperature stable α-phase. Depending on temperature and heating/cooling rates, a variety of α/β morphologies can form that gives varying mechanical properties. The complex relationship between thermomechanical-processing, microstructure and mechanical properties was investigated by References [28,29]. On the basis of the literature [30,31,32,33], few microstructural features have been identified as relevant concerning mechanical properties. The three separate α-phase fractions, Widmanstatten ($X_{\alpha_{w}}$), grain boundary ($X_{\alpha_{gb}}$), acicualr and massive Martensite ($X_{\alpha_{m}}$) and β-phase fraction ($X_{\beta }$) were included in the current model. Though in the current flow-stress model individual α-phase fractions were not included separately, it is possible to incorporate them when more details about their respective strengthening mechanisms are known.

### 3.1. Phase Transformations

**Figure 1:**
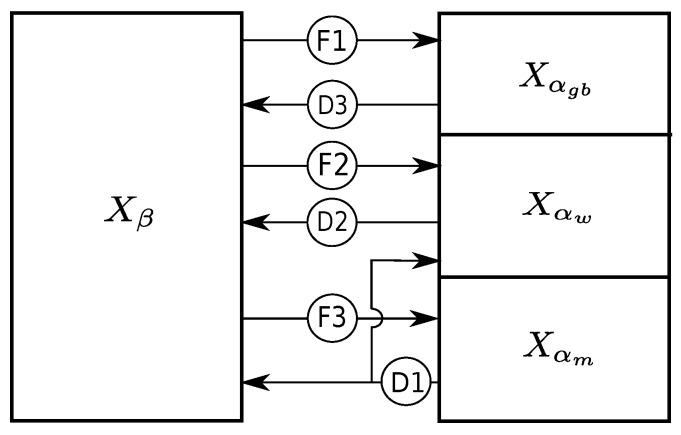
Phase-change mechanism.

**Figure 2:**
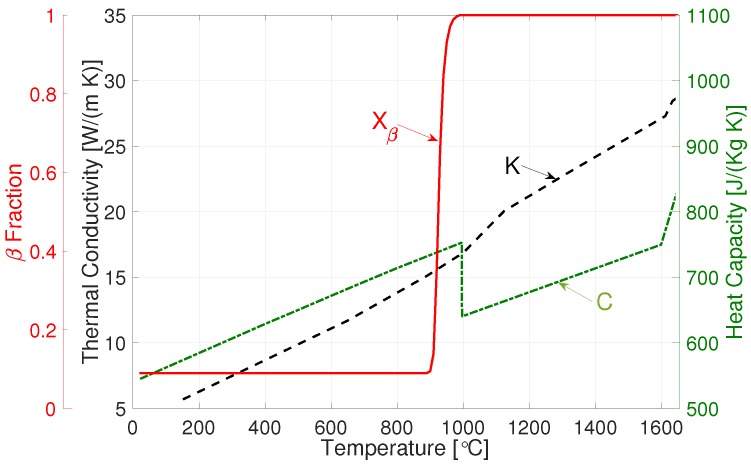
Thermal conductivity (K), specific heat capacity (C) and equilibrium-phase fraction ($X_{\beta }$).

Depending on the temperature and heating/cooling rates, Ti-6Al-4V undergoes allotropic transformation. The mathematical model for the transformation is described schematically in Figure 1. Transformations denoted by F1, F2, and F3 represent the formation of $\alpha_{gb}$, $\alpha_{w}$, and $\alpha_{m}$ phases, respectively, and D3, D2 and D1 show the dissolution of the same phases. If the current volume fraction of β phase is more than $\beta_{eq}$, the excess β phase transforms into an α phase. Here, $\alpha_{gb}$ formation that occurs in high temperatures is the most preferred, followed by the $\alpha_{w}$. Remaining excess β fraction is transformed to $\alpha_{m}$ if the temperature is lower than $T_{m}$, (martensite start temperature) and cooling rate is above 20 °C/s. Conversely, if the current volume fraction of β is lower than $\beta_{eq}$, excess α phase is converted to β. Primarily, the $\alpha_{m}$ phase dissolves to β and $\alpha_{w}$ phases in the same proportion as the $\alpha_{eq}$ and $\beta_{eq}$. Remaining excess $\alpha_{w}$ and $\alpha_{gb}$ transform to β in that order. The equilibrium fraction of the β phase (see Figure 2) is computed by Equation (17), where *T* is the temperature in degrees Celsius.

(17) $$\begin{array}{ccc} X_{\beta }^{eq} & = & 1-0.89\;e^{{-\left(\frac{T^{*}+1.82}{1.73}\right)}^{2}}+0.28\;e^{{-\left(\frac{T^{*}+0.59}{0.67}\right)}^{2}}\\ T^{*} & = & (T-927)/24. \end{array}$$

### 3.2. Adaptation of Johnson–Mehl–Avrami–Kolmogrov (JMAK) Model for Diffusional Transformation

The JMAK model [34,35,36], originally formulated for nucleation and growth during isothermal situations, can be adapted to model any diffusional transformation. Employing the additivity principle and using sufficiently small time steps ensures that any arbitrary temperature change can be computed. The JMAK model assumes that a single phase $X_{1}$ that is 100% in volume from the start transforms to 100% of second phase $X_{2}$ in infinite time. However, in the case of Ti-6Al-4V, this is not the case, as it is a α−β dual-phase alloy below β-transus temperature. Hence, in order to accommodate for an incomplete transformation, the product fraction is normalized with the equilibrium volume. Conversely, the starting volume of a phase can also be less than 100%, which is circumvented by assuming that the available phase volume is the total phase fraction. Another complication is the existence of the simultaneous transformation of various α phases ($\alpha_{w}$, $\alpha_{gb}$, $\alpha_{m}$) to the β phase and back. This can be modelled by sequentially calculating each transformation within the time increment [27].

### 3.3. Formation of α Phase

During cooling from the β-phase, the $\alpha_{gb}$ and $\alpha_{w}$ phases are formed by diffusional transformation. According to the incremental formulation of the JMAK model described by Reference [27], the formation of $\alpha_{gb}$ and $\alpha_{w}$ can be modelled by the set of equations in rows F1 and F2, respectively, of Table 1. The Martensite phase is formed at cooling rates above 410 °C/s by diffusion-less transformation. While cooling at rates above 20 ${}^{\circ }$C/s and up to 410 ${}^{\circ }$C/s, massive α transformation was observed to co-occur with Martensite formation [37,38]. Owing to the similitude in crystal structures between massive-α and Martensite-α, they are not differentiated here except that, above 410 °C/s, 100% $\alpha_{m}$ was allowed to form. An incremental formulation of the Koistinen–Marburger equation described by Charles Murgau et al. [27] was used here (see equation set in row F3 of Table 1).

### 3.4. Dissolution of α Phase

The $\alpha_{m}$ phase formed by instantaneous transformation is unstable and therefore undergoes diffusional transformation to the $\alpha_{w}$ and β phases on the basis of its current equilibrium composition. The incremental formulation of the classical JMAK model by Reference [27] and its parameters are given in row D1 of Table 2. During heating or reaching nonequilibrium phase composition, $\alpha_{w}$ and $\alpha_{gb}$ can transform into a β-phase controlled by the diffusion of vanadium at the α−β interface. A parabolic equation developed by Kelly et al. [39,40] derived in its incremental form by Charles Murgau et al. [27] was used here (see rows D2 and D3 of Table 2).

## 4. Coupling of Phase and Flow-Stress Models

**Figure 3:**
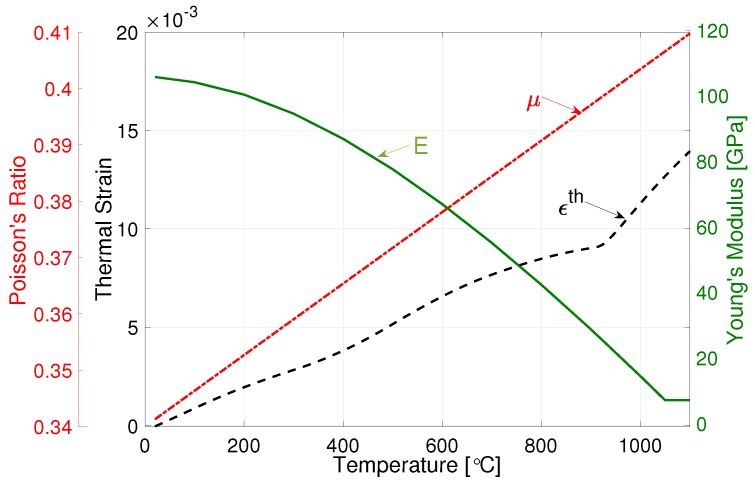
Poisson’s ratio (μ), thermal strain ($\epsilon^{th}$) and Youngs modulus (E).

Young’s modulus and Poisson’s ratio were assumed to be identical for both phases. The Wachtman model [41] for Young’s modulus (*E*), calibrated using measurements from Babu and Lindgren [11], is written as

(18) $$E=107-0.2\;\left(T+273\right)\;e^{-\left(MM1300\;/\;T+273\right)},$$

where *T* is the temperature in degrees Celsius applied with a cut-off at *T* = 1050 °C (see Figure 3). A linear model for Poisson’s ratio (μ) after fitting to measurements by Fukuhara and Sanpei [42] as

(19) $$\begin{array}{ccc} \mu & = & 0.34+6.34\;e^{-5}\;T, \end{array}$$

where *T* is the temperature in degrees Celsius (see Figure 3).

Using X-ray diffraction, Swarnakar et al. [43] measured the volumetric expansion of unit cells of α and β phases during heating. On this basis, the average Coefficient of Thermal Expansion (CTE) of the phase mixture can be calculated using the rule of mixtures (ROM) as in Equation (20), where $\alpha_{\alpha }$ and $\alpha_{\beta }$ give the CTE of α and β phases, respectively. The linear thermal strain can be computed using Equation (21), plotted in Figure 3. Here, $\varepsilon^{adj}$ makes the ROM (Equation (20)) nonlinear.

(20) $$\begin{array}{ccc} \alpha_{avg} & = & X_{\alpha }\alpha_{\alpha }+X_{\beta }\alpha_{\beta } \end{array}$$

(21) $$\begin{array}{ccc} \varepsilon^{th} & = & \alpha_{avg}\Delta T-\varepsilon^{adj} \end{array}$$

(22) $$\begin{array}{ccc} \varepsilon^{adj} & = & 1.0e^{-8}T^{2}-8.4e^{-6}T+3.0e^{-4}. \end{array}$$

The thermal conductivity and specific heat capacity of the alloy taken from References [44] and [45], respectively, are given in Figure 2. The latent heat of phase transformation $\left(\alpha \to \beta \right)$ and the latent heat of fusion were measured to be 64 and $\left(290\pm 5\right)$ kJ/Kg, respectively [44].

The yield strength of the phase mixture can be written according to the linear rule of mixtures as

(23) $$\sigma_{y}=X_{\alpha }\sigma_{y}^{\alpha }+X_{\beta }\sigma_{y}^{\beta }.$$

The plastic strain distribution can be obtained assuming an iso-work principle. According to Reference [46], this can be written as

(24) $$\begin{array}{ccc} \sigma_{y}^{\alpha }{\dot{\bar{\varepsilon }}}_{\alpha } & = & \sigma_{y}^{\beta }{\dot{\bar{\varepsilon }}}_{\beta } \end{array}$$

(25) $$\begin{array}{ccc} {\dot{\bar{\varepsilon }}}^{p} & = & X_{\alpha }{\dot{\bar{\varepsilon }}}_{\alpha }+X_{\beta }{\dot{\bar{\varepsilon }}}_{\beta }. \end{array}$$

**Figure 4:**
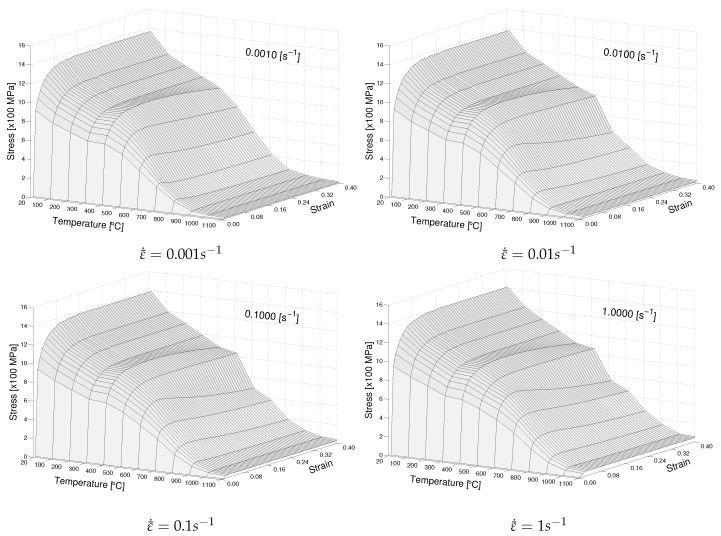
Stress–strain–temperature relationship.

The above formulation ensures that the β phase with lower yield strength has a more significant share of plastic strain as compared to the stronger α phase. For temperatures above 1100 °C, ($\sigma_{y}^{>1100{\;}^{\circ }C}=\sigma_{y}^{1100{\;}^{\circ }C}$). The stress–strain relationship predicted by the model for varying strain rates and temperatures are given in Figure 4. The rate dependence and flow-softening demonstrated by the model is visible here. A detailed comparison of model predictions and measurements along with model parameters are given in Babu et al. [10,11].

## 5. Additive Manufacturing

In this article, a DED process described in Reference [47] was simulated using general-purpose Finite Element (FE) software MSC.Marc. A set of subroutines for modelling the AM process were implemented in MSC.Marc, which are explained in Lundbäck and Lindgren [48]. A coupled thermomechanical–metallurgical model described in the previous sections was also implemented as subroutines within MSC.Marc.

**Figure 5:**
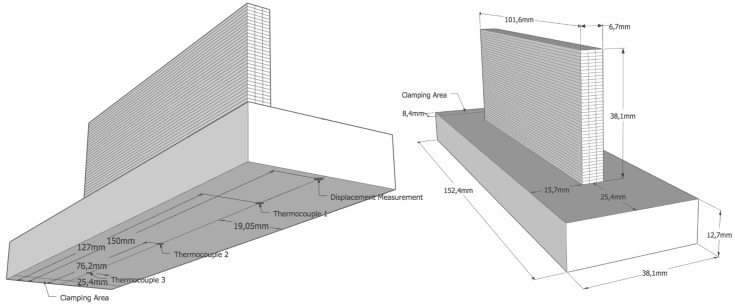
Dimensions of additive-manufacturing (AM) component.

The dimensions of the substrate (152.4 × 38.1 × 12.7 mm) and AM component are given in Figure 5. One end of the fixture was held in position by a clamping fixture (see notations in Figure 5).

**Figure 6:**
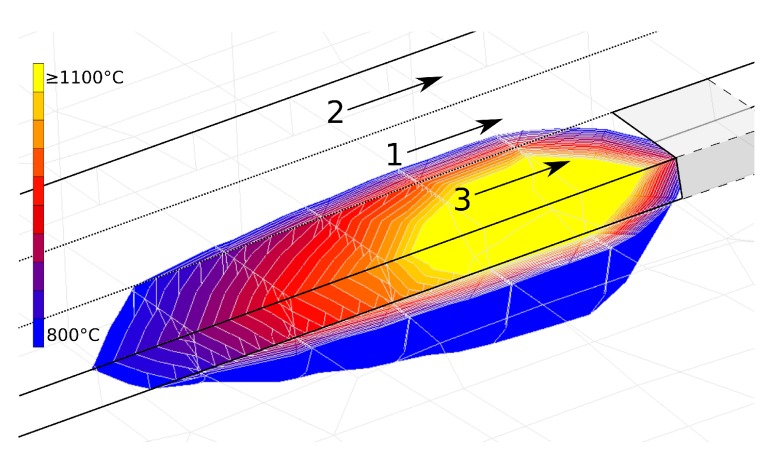
Temperature contours in weld pool.

Three beads were added per layer with a total width of 6.7 mm and a height of 38.1 mm; 42 discrete layers and their respective beads are also shown in Figure 5. Figure 6 shows the order of deposition starting with the middle bead, followed by one on each side. Odd layers are deposited in the direction away from the clamping, followed by even layers in the opposite direction. Figure 6 also shows the temperature contours and direction of deposition of bead three of the first layer. After each layer, a dwell time (DT) of 0, 20 and 40 s was applied for studying the effect of varying cooling rates.

## 6. Modelling of Additive Manufacturing

**Figure 7:**
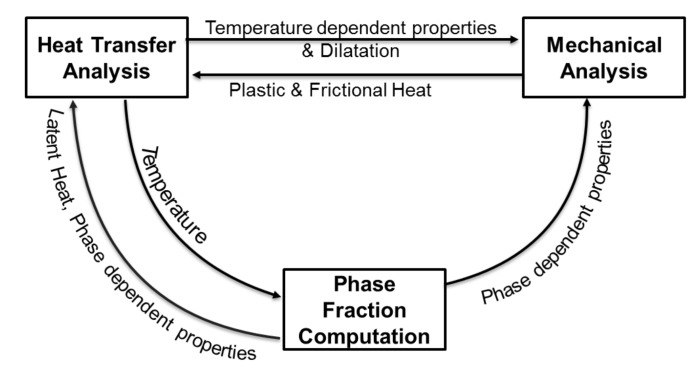
Coupling of thermomechanical–microstructural fields.

In the current work, the scope of the model was to predict microstructure evolution, the overall distortion of the component and residual stresses. This requires a solution where thermomechanical–metallurgical models are coupled by using a staggered approach. Figure 7 shows the coupling of different domains using a staggered approach. The thermal field is solved using the FE implicit iterative scheme by computing heat input and heat losses by conduction, convection and radiation. On the basis of the computed temperature in the increment, the metallurgical model computes the phase evolution for each Gauss point. The computed temperature and phase fractions are input to solve the mechanical-field equations. A large-deformation FE implicit iterative scheme was used here, where mechanical and physical properties are strongly dependent on the temperature and phase composition. Latent heat and volume changes due to phase evolution and deformation energy converted to heat are included here.

### 6.1. Heat Source

**Figure 8:**
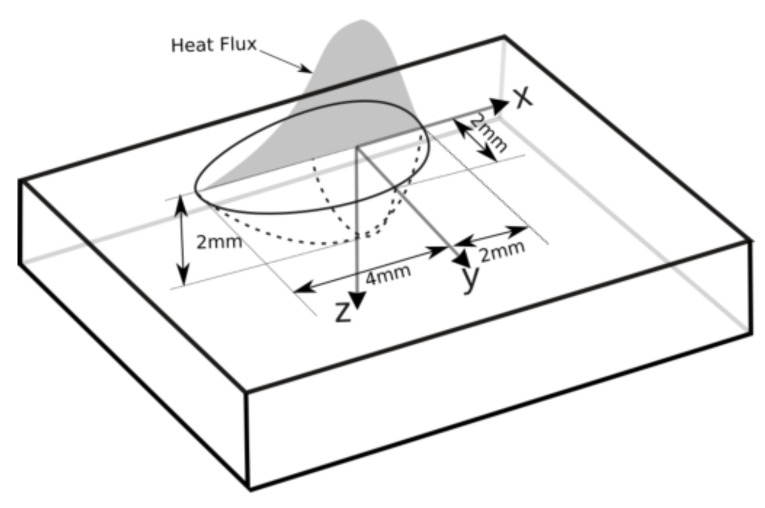
Gaussian distribution of density and double ellipsoid shape in xy plane.

Modelling of weld-pool phenomena requires high-resolution discretization and at least one other physics domain, namely, fluid flow. This requires an impractical amount of resources for solving the problem and can be avoided considering the scope of the current work. The heat input can be modelled by using volume heat flux in a geometric region representing the weld pool, and is calibrated using measured temperatures. Goldak’s [49] double ellipsoidal heat-input model was implemented in the current work, with efficiency calibrated to be 0.29. The parameters of the heat source are given in Figure 8. See Lundbäck and Lindgren [48] for details on the implementation of this model.

### 6.2. Modelling of Material Addition

The inactive-element approach was used here, where all elements representing the added metal were deactivated before the start of the simulation, and only activated after meeting certain criteria. In each increment, the set of elements that overlapped and the geometric region represented by the current weld-pool position were thermally activated, whereas mechanical activation occurred only when the thermally active elements cooled below the solidus temperature. Before thermal activation, the elements may have had to be moved to accommodate for the distortion of the substrate and the already activated elements during the process. The moving elements maintained connectivity with the activated elements, and their volume matched the added material during that time step [48].

### 6.3. Boundary Conditions

In DED, much of the heat input in the initially deposited layers is absorbed by the substrate. To balance the heat input, losses by free and forced convection, and conduction to fixtures as well as radiation were included in the model. A lumped convective coefficient of 18 W/m${}^{2}/$°C was applied to model the natural and forced convection from shielding gas. Both convective and radiative boundary conditions were applied on the outer surface of all thermally active elements. A surface emissivity of 0.25 was used here. In the current model, heat losses due to cooling by the fixture were achieved by using convective heat transfer with a high coefficient of 250 W/m${}^{2}/$°C.

## 7. Comparison of Measurements and Simulations

**Figure 9:**
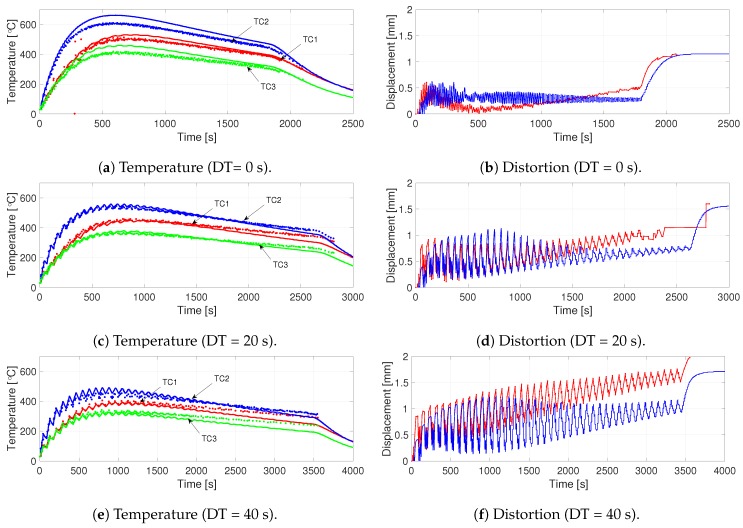
Comparison of Measurements and Simulations.

In situ measurement of temperature and distortion was done during the AM process. Three thermocouples were attached to the bottom of the substrate at the positions shown in the left part of Figure 5. DTs allowed the component to considerably cool down during the process. In Figure 9a,c,e, dots denote the thermocouple measurements and lines, predictions from the model. The thermocouple attached to the middle of the component (TC2) registered the highest temperature, as the other two were closer to the ends that are subjected to higher convective cooling. The thermocouple attached close to the clamping (TC3) had the lowest temperature since the fixture acts as a heat sink. Raising dwell times by 20 s increased cooling, thereby reducing the peaks. As the height of the wall increased, this effect was less detectable, as this thermocouple was beneath the substrate.

**Figure 10:**
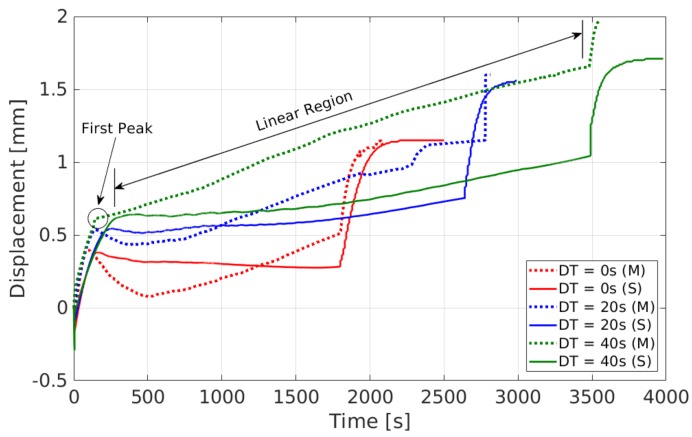
Comparison of distortions.

The component distortion is measured at the free end by using a laser displacement sensor. In Figure 9b,d,f, red lines denote the measured values, and the blue lines denote predictions from the model. The addition of each layer made the component bend downwards due to the thermal gradient between the top and bottom of the substrate, which is diminished during cooling, producing oscillations. In order to compare measurement and simulation results, these oscillations were smoothed out by using the Savitzky–Golay filter [50], and are plotted in Figure 10. Here, dotted lines denote measurements, and continuous lines denote model predictions. The start and end of the linear region, its slope and the detection of the first peak can be deduced from Figure 10 and are shown in Table 3. The peak-to-peak amplitude of the oscillations at the first peak, and also at the start and end of the linear region are given in Table 3. The measured final bending of the build plate at the outer edge increased by 0.4 mm for each 20 s increase in dwell time. Simulations also gave a similar result. Simulation results are given in Table 3 within brackets.

**Figure 11:**
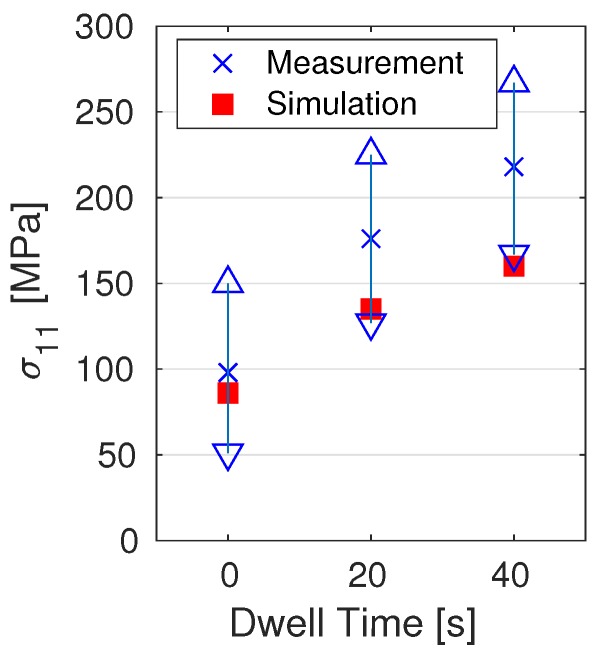
Residual stress.

**Figure 12:**
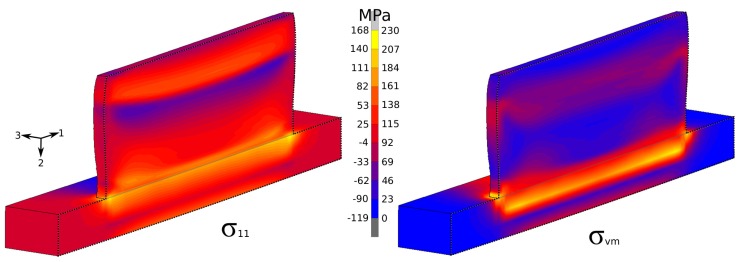
Residual stress for 0 s dwell time (model clipped longitudinally in midplane).

**Figure 13:**
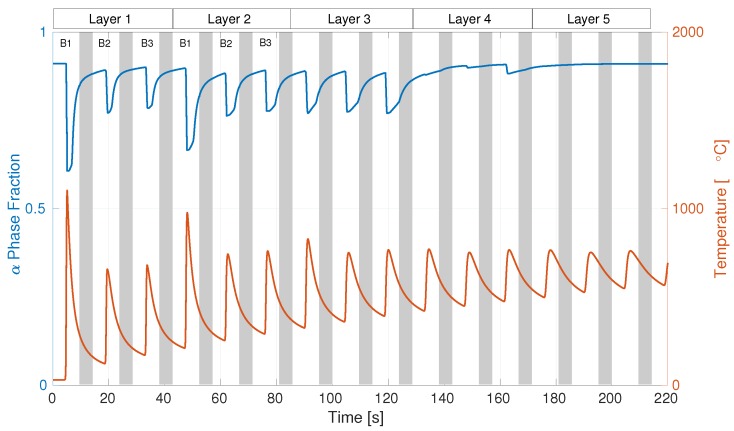
Prediction of phase fraction and temperature.

Figure 11 shows the residual stress in the welding direction along with its spread measured by Reference [47] by using hole drilling. The testing location was in the middle of the specimen at the bottom of the substrate. Results showed that, for the case with dwell times of 0 and 20 s, simulation results were close to the measurements or within the margin of error; for 40 s, it was slightly below the margin. Residual-stress distribution after cooling to room temperature in the welding direction and the von Mises effective stress for the case with dwell time of 0 s are plotted in Figure 12. The predicted temperature for the case with dwell time of 0 s at the top surface of the substrate above the location of TC2 is given in Figure 13. The computed α-phase fraction is also provided here. The addition of each bead is denoted as B1-3, and grey areas in between are the cooling time. In total, five layers are shown in Figure 13.

## 8. Discussion

The yield strength of the material is very much dependent on state variables of dislocation density and excess vacancy concentration. The density of these state variables changes by many orders of magnitude during heating and deformation. Deformation increases dislocation density and results in hardening, whereas an increase in vacancy concentration (due to heating and deformation) assists in the remobilization of dislocations, thereby material recovery. The advanced material model described here combining the metallurgical and flow-stress models has proven to be suitable for AM simulation. Diffusional and instantaneous transformations are included in the metallurgical model. This model was formulated in a way that it could be implemented in any standard kind of finite-element software. Temperature measurements and results from the simulations demonstrated a good overall fit. This model predicted the final distortion of the component with good accuracy except for the case with 40 s dwell time. This trend was also evident in the residual-stress measurements. The comparison of distortion before the onset of cooling showed a larger difference between model and measurements. This might be because the stress-relaxation behavior was less accurately predicted by the model. The computed phase fraction in Figure 13 showed that, after the addition of the fourth layer, the substrate underwent no significant phase evolution. Temperature peaks after 120 s had slightly less magnitude, and were therefore below the cutoff levels to introduce any phase change. Denlinger and Michaleris [51] performed the simulation of all the three cases described here. They used an approach where the plastic strain was reset to zero at a temperature of 690 °C which is a parameter calibrated for that particular AM case. This transformation-strain parameter made it possible for Denlinger and Michaleris [51] to include the effects of dwell time, whereas in the current work, mechanisms of dislocation climb and globularization resulted in the restoration of the lattice.

## 9. Conclusions

One of the challenges involved in the AM process is residual deformation and stresses due to the thermal dilatation of the substrate and added structure. The final properties of the AM structure are strongly influenced by the microstructure, which is dependent on the thermomechanical-processing history of the component. For the industry to fully adopt additive manufacturing and be able to qualify titanium parts for critical applications, such as in aerospace, a complete understanding of the microstructure properties and mechanical behavior is necessary. This paper showed the implementation and application of a coupled microstructural–thermal–mechanical model to an AM process. A physically based constitutive model was explicitly coupled to the microstructural model. The phase composition predicted by the microstructural model therefore affected the mechanical properties, namely, flow strength, in a direct way. Validation of the thermal and mechanical model was performed by comparing the simulation results with the available measurements in the literature. The comparison had good agreement between the results from the model and the measurements.

## Figures and Tables

**Table 1:** Models for α-phase formation.

F1	${}^{n+1}X_{\alpha_{gb}}=\left(1-e^{-k_{gb}{\left(t_{gb}^{*}+\Delta t\right)}^{N_{gb}}}\right)\left({}^{n}X_{\beta }+{}^{n}X_{\alpha w}+{}^{n}X_{\alpha_{gb}}\right){}^{n+1}X_{\alpha }^{eq}-{}^{n}X_{\alpha_{w}}$
	$t_{gb}^{*}=\sqrt[{N_{gb}}]{-ln\left(1-\frac{\left({}^{n}X_{\alpha w}+{}^{n}X_{\alpha_{gb}}\right)/{}^{n+1}X_{\alpha }^{eq}}{{}^{n}X_{\beta }+{}^{n}X_{\alpha w}+{}^{n}X_{\alpha_{gb}}}\right)/k_{gb}}$
F2	${}^{n+1}X_{\alpha_{w}}=\left(1-e^{-k_{w}{\left(t_{w}^{*}+\Delta t\right)}^{N_{w}}}\right)\left({}^{n}X_{\beta }+{}^{n}X_{\alpha_{w}}+{}^{n}X_{\alpha_{gb}}\right){}^{n+1}X_{\alpha }^{eq}-{}^{n}X_{\alpha_{gb}}$
	$t_{w}^{*}=\sqrt[{N_{w}}]{-ln\left(1-\frac{\left({}^{n}X_{\alpha_{w}}+{}^{n}X_{\alpha_{gb}}\right)/{}^{n+1}X_{\alpha }^{eq}}{{}^{n}X_{\beta }+{}^{n}X_{\alpha_{w}}+{}^{n}X_{\alpha_{gb}}}\right)/k_{w}}$
F3	${}^{n+1}X_{\alpha_{m}}=\left\{\begin{array}{c} \left(1-e^{-b_{km}}\left(T_{ms}-T\right)\right)\left({}^{n}X_{\beta }+{}^{n}X_{\alpha_{m}}\right);\;if\;\left(\dot{T}\;>\;410{\;}^{\circ }C/s\right)\\ \left(1-e^{-b_{km}}\left(T_{ms}-T\right)\right)\left({}^{n}X_{\beta }+{}^{n}X_{\alpha_{m}}-{}^{n+1}X_{\alpha }^{eq}\right);\;if\;\left(20{\;}^{\circ }C/s\;>\;\dot{T}\;>\;410{\;}^{\circ }C/s\right) \end{array}\right.$

**Table 2:** Models for α-phase dissolution.

D1	${}^{n+1}X_{\alpha_{m}}=\left({}^{n+1}X_{\alpha_{m}}^{eq}-e^{-k_{m}{\left(t_{m}^{*}+\Delta t\right)}^{N_{m}}}\right)\left({}^{n}X_{\beta }+{}^{n}X_{\alpha_{m}}-{}^{n+1}X_{\alpha_{m}}^{eq}\right)$
	$t_{m}^{*}=\sqrt[{N_{m}}]{-ln\left(\frac{\left({}^{n}X_{\alpha }-{}^{n+1}X_{\alpha_{m}}^{eq}\right)}{{}^{n}X_{\beta }+{}^{n}X_{\alpha_{m}}-{}^{n+1}X_{\alpha_{m}}^{eq}}\right)/k_{m}}$
D2	${}^{n+1}\left(X_{\alpha_{w}}+X_{\alpha_{gb}}\right)=\left\{\begin{array}{c} {}^{n+1}X_{\alpha }^{eq}f_{diss}\left(T\right)\sqrt{\Delta t+t^{*}};\;if\;\left(0\;<\;\left(\Delta t+t^{*}\right)\;<\;t_{crit}\right)\\ {}^{n+1}X_{\alpha }^{eq};\;if\;\left(\Delta t+t^{*}\;>\;t_{crit}\right) \end{array}\right.$
D3	$t^{*}={\left(\frac{{}^{n}X_{\beta }}{{}^{n+1}X_{\beta }^{eq}f_{diss}\left(T\right)}\right)}^{2}$

**Table 3:** Comparison of distortion (computed values given in brackets).

	0 s (comp)	20 s (comp)	40 s (comp)
Final displacement [mm]	1.2 (1.2)	1.6 (1.6)	2.0 (1.7)
Slope of linear region [10${}^{-4}$mm/s]	3.4 (−0.2)	3.7 (1.1)	3.2 (1.4)
Time at 1st Peak [s]	110 (140)	170 (230)	150 (300)
Amplitude at 1st Peak [10${}^{-1}$mm]	3.5 (3.8)	7.6 (7.0)	6.4 (6.9)
Amplitude at start of linear region [10${}^{-1}$mm]	1.0 (0.9)	6.2 (8.4)	6.6 (6.1)
Amplitude at end of linear region [10${}^{-1}$mm]	0.1 (0.4)	1.1 (0.8)	2.4 (2.1)

## Abbreviations

The following abbreviations are used in this manuscript:

DED	Directed Energy Deposition
TMM	Thermomechanical-Microstructural
AM	Additive Manufacturing
JMAK	Johnson–Mehl–Avrami–Kolmogrov
PBF	Powder Bed Fusion
FE	Finite Element
ROM	Rule of Mixtures
CTE	Coefficient of Thermal Expansion
CAD	Computer Aided Design
CNC	Computer Numerical Controlled
DT	Dwell Time
TC	Thermocouple
